# Induced expression modes of genes related to Toll, Imd, and JAK/STAT signaling pathway-mediated immune response in *Spodoptera frugiperda* infected with *Beauveria bassiana*

**DOI:** 10.3389/fphys.2023.1249662

**Published:** 2023-08-24

**Authors:** Jihu Li, Yongkai Mao, Jiequn Yi, Mingjiang Lin, Hanliang Xu, Yinjie Cheng, Han Wu, Jianbai Liu

**Affiliations:** Institute of Nanfan and Seed Industry, Guangdong Academy of Sciences, Guangzhou, China

**Keywords:** *Spodoptera frugiperda*, *Beauveria bassiana*, transcriptome, signaling pathways, gene expression

## Abstract

*Spodoptera frugiperda* is one of the most harmful pests that attack maize and other major food crops and causes huge economic loss every year in China and other countries and regions. *Beauveria bassiana*, a kind of entomological fungus that is highly pathogenic to pests, is harmless to the environment and human beings. However, at present, *S. frugiperda* has gradually developed resistance to many pesticides and microbial insecticides. In this study, transcriptome sequencing was conducted to analyze the differences in gene expression between *B. bassiana*-infected and -uninfected *S. frugiperda*. More than 160 Gb of clean data were obtained as 150-bp paired-end reads using the Illumina HiSeq™ 4000 platform, and 2,767 and 2,892 DEGs were identified in LH36vsCK36 and LH144vsCK144, respectively. In order to explore the roles of JAK/STAT, Toll, and Imd signaling pathways in antifungal immune response in *S. frugiperda* against *B. bassiana* infection, the expression patterns of those signaling pathway-related genes in *B. bassiana*-infected *S. frugiperda* were analyzed by quantitative real-time PCR. In addition, antifungal activity experiments revealed that the suppression of JAK/STAT, Toll, and Imd signaling pathways by inhibitors could inhibit the antifungal activity to a large extent and lead to increased sensitivity of *S*. *frugiperda* to *B. bassiana* infection, indicating that JAK/STAT, Toll, and Imd signaling pathways and their associated genes might be involved in the synthesis and secretion of antifungal substances. This study implied that JAK/STAT, Toll, and Imd signaling pathways played crucial roles in the antifungal immune response of the *S. frugiperda* larvae, in which the related genes of these signaling pathways could play special regulatory roles in signal transduction. This study would improve our understanding of the molecular mechanisms underlying innate immunity and provide the basis for a wide spectrum of strategies against antifungal resistance of *S. frugiperda*.

## Introduction

The immune mechanism of insects belonging to the invertebrate phylum *Arthropoda* (*Insecta*) mainly relies on humoral and cellular immunity. The humoral immunity of insects is mainly composed of antimicrobial peptides and lysozymes against pathogenic fungi and antiviral factors, lectins, and other immune factors against viruses and other pathogens (Lemaitre and Hoffmann, 2007). The cellular immunity of insects is mainly based on the ability of blood cells (hemocytes) of insects, such as granule cells, plasma cells, bead cells, and granulocytes, to recognize and phagocytose pathogenic microorganisms and heal the wound of insects (Kanost et al., 2004; Rao and Yu, 2010).

There are four signaling pathways {JAK/STAT (Janus kinase/signal transducer and activator of transcription) signaling pathway, Toll signaling pathway, Imd (immunodeficiency) signaling pathway, and JNK (c-Jun NH (2)-terminal protein kinase) signaling pathway} in insects that mediate the immune response to different pathogens. The Toll signaling pathway and Imd signaling pathway are classical immune signaling pathways that mediate the transcription and synthesis of most antimicrobial peptides (AMPs) and other active substances (Tanji et al., 2007).

The JAK/STAT signaling pathway is guided by a variety of cytokines and involved in the regulation of many important physiological processes, including growth regulation (Hirano et al., 2000), cell proliferation and differentiation, cell apoptosis, embryonic development (Pires-daSilva and Sommer, 2003), and innate regulation of immunity (Brivanlou et al, 2002a; Karst et al., 2003; Souza-Neto et al., 2009). The extracellular binding of peptide ligands to specific transmembrane receptors initiates the activation of the JAK/STAT signaling pathway. The receptor undergoes conformational transformation and self-phosphorylation by receptor-related Janus kinases, and activated JAKs, in turn, phosphorylate the receptor, which favors the formation of docking sites for cytoplasmic signal transducer and activator of transcription (STAT). Then, the phosphorylated STATs, under the action of JAK, eventually translocate to the nucleus and activate the transcription of target genes (Levy and Darnell, 2002; Li, 2008; Zhang et al., 2023).

In general, the Toll signaling pathway can rapidly mediate the synthesis and secretion of antifungal and antibacterial peptides (Tauszig et al., 2000). When pathogens are recognized, the activated serine protease cascade also activates the Toll signaling pathway. After entering cells, immune signals complete the cytosolic signal transduction process through the core signal factors of the Toll signaling pathway and finally transduce into the nucleus and initiate the expression of target genes (Imler and Hoffmann, 2001; Tauszig-Delamasure et al., 2002; Naitza and Ligoxygakis, 2004; Uvell and Engström, 2007; Ao et al., 2008). The Imd signaling pathway is another important signaling pathway involved in the regulation of the innate immune system, which was first identified and characterized in *Drosophila* (Corbo and Levine, 1996; Rämet et al., 2002). The Imd signaling pathway mainly regulates antimicrobial peptides, such as aggresin, cecropin, drosocin, and diptericin, that exert effects on Gram-negative bacteria (Levashina et al., 1998; Kaneko et al., 2004a). After Gram-negative bacteria are recognized, the activated Imd proteins act on transcription growth factor kinase 1 (TAK1), which transmits immune signals to IKKs (including IKK-β and IKK-γ) (Rutschmann et al., 2000; Silverman et al., 2003). Then, IKKs activate Relish in two ways. One is that IKKs directly phosphorylate the NF-κB-like signaling factor Relish and activate it (Silverman et al., 2000). Another way is that IKKs function on the DREDD–Fadd–Relish complex, which hydrolyzes and releases the activated Relish protein (Leulier et al., 2002). Relish has two homologs, Relish1 and Relish2, which are activated and then enter the nucleus to regulate the transcription of antimicrobial peptide-related target genes downstream. The cellular immunity of insects mainly involves recognition and phagocytosis of pathogenic microorganisms by insect blood cells, such as granulosa cells, plasma cells, bead cells, and granulocytes, as well as the wound healing of insect bodies.

*Spodoptera frugiperda*, a lepidopteran of the family *Noctuidae*, originates from tropical and subtropical areas of the Americas (Todd and Poole, 1980). It is one of the most dangerous agricultural pests in the world and causes serious damage to maize and other major food and economic crops every year (Martinelli et al., 2006). *S. frugiperda* not only has an adverse impact on social economy and food security but also leads to a significant increase in the use of highly toxic pesticides. The feeding characteristics and development of resistance of *S. frugiperda* have led to the reuse and overuse of pesticides; these two interrelated problems potentially give rise to food crises and human and environmental health problems (Prasanna et al., 2018). In recent years, the abuse of chemical pesticides has led to a series of problems, such as drug resistance, excessive pesticide residues, and environmental pollution. Compared with conventional chemical control, microbial control has the advantages of specificity and ecological safety. Microbial pesticides, such as bacteria, fungi, and viruses, play an important role in the control of *S. frugiperda*. At present, entomopathogenic microorganisms such as *Beauveria bassiana*, *Bacillus thuringiensis*, and nuclear polyhedrosis viruses (SfMNPVs) have been registered and utilized for the control of *S. frugiperda* both at home and abroad (Poisot et al., 2018). *B. bassiana* is a kind of entomological fungus, which can infect more than 700 types of insects belonging to 149 families and 15 orders. At the same time, *B. bassiana* is harmless to the environment and warm-blooded animals but has a strong virulence for pests; its culture conditions are relatively simple; and the strain is cheap and easy to obtain. Currently, this pathogenic fungus is widely used as a biopesticide to control pests around the world (Ramos et al., 2017).

However, there are few reports on the immune response genes related to signaling pathways of *S. frugiperda*, an invasive pest in China, after its infection with *B. bassiana*. Therefore, in this study, high-throughput transcriptome sequencing was performed on *S. frugiperda* larvae collected at 36 h and 144 h after *B. bassiana* infection to screen out the genes related to the immune response of *S. frugiperda* to *B. bassiana* infection. This study would provide target genes for improving the control effect of *B. bassiana* against *S. frugiperda* and lay a foundation for understanding the mechanism of resistance of *S. frugiperda* to *B. bassiana*.

## Materials and methods

### Insects and tested strains

The larvae of *S. frugiperda*, obtained from a wild field, were reared at 27°C ± 1°C with a relative humidity of 65%∼85% and a photoperiod of 14 L:10 D at the Institute of Nanfan and Seed Industry, Guangdong Academy of Sciences, Guangzhou, China. After at least three generations, the third-instar larvae with the same individual length and instar and moderate epidermal state were selected as the experimental subjects.

### Preparation of *B. bassiana* conidial suspension

*B. bassiana* was cultured on a potato dextrose agar (PDA) medium at 27°C ± 1°C for 1 week. Then, 10 ml 0.15 mol/L NaCl solution was added into the tubes which were then oscillated gently. The conidia and hyphae were transferred into a centrifuge tube with 20 μL Tween-80, vortex-shaken for 10 min, and then filtered. The spores were counted using a blood count plate and diluted with 0.15 mol/L NaCl solution to a concentration of 10^8^ spores/ml. Then, 1 mL of the prepared conidia suspension was added into 10 mL potato dextrose (PD) medium and then cultured at 30°C ± 1°C, 180 rpm for 3 days. The mixture of hyphae and blastospores were autoclaved at 121°C for 20 min to generate the heat-inactivated suspension of *B. bassiana*. Finally, the *B. bassiana* conidial suspension and inactivated *B. bassiana* conidial suspension were used for immune induction in *S. frugiperda*.

### cDNA library construction, transcriptome sequencing, assembly, and functional annotation

The larvae of *S. frugiperda* were treated with 1 × 10^8^/mL spore suspension of *B. bassiana* Bb378, while those treated with 0.05% Tween-80 solution were set as controls. The infected larvae were collected at 36 and 144 h after treatment, then flash-frozen with liquid nitrogen, and stored at −80°C. Each treatment was performed using three biological replicates. For every sample, the total RNA was extracted using TRIzol reagent (Invitrogen, United States) according to the manufacturer’s instructions. Contaminating genomic DNA was removed using RNase-free DNase I (TaKaRa Biotechnology Co., Ltd., Dalian, China), and then, the quantity and quality of RNA were assessed. RNA with high concentration, integrity, and purity was chosen for cDNA library construction and final Illumina sequencing at Novogene Bioinformatics Technology Co., Ltd. (Beijing, China). The obtained cDNA was then tested and sequenced on the Illumina HiSeq™ 4000 platform as 150-bp paired-end reads.

The adapters, primers, ambiguous “N” nucleotides, and low-quality (50% of the bases had a quality value ≤5) sequences were removed from the raw data to obtain clean reads. Then, the quality of the clean reads was assessed by Q30 (percentage of bases with a Phred value of at least 30), the GC content, and sequence duplication level. The clean data were assembled into *de novo* contigs using Trinity software (Grabherr et al., 2011). Subsequently, transcripts were assembled and obtained by using the de Bruijn graph method. Finally, unigenes were formed from the assembled transcripts using the TGI Clustering tool (Quackenbush et al., 2001; Pertea et al., 2003).

Annotations of all unigenes were performed using BLASTx against a pooled database of the National Center for Biotechnology Information non-redundant (NCBI-NR) protein, UniProt, Gene Ontology (GO), and Kyoto Encyclopedia of Genes and Genomes (KEGG) with an E-value <10^−5^. The annotation of unigenes was obtained using HMMER software (Eddy, 1998), and then, Gene Ontology (GO) annotations were performed on all unigenes using Blast2GO. Subsequently, WEGO was used to determine GO functional classification and evaluate the distribution of gene functions at the macro-level (Ye et al., 2006). Metabolic pathway annotations for the unigenes were predicted based on the KEGG annotations (Kanehisa et al., 2008).

### Expression analysis by real-time quantitative PCR

The expression of 28 tested genes related to the Toll signaling pathway, Imd (immunodeficiency) signaling pathway, and JAK/STAT (Janus kinase/signal transducer and activator of transcription) signaling pathway was verified by real-time quantitative PCR (qRT-PCR) with specific primers (Supplementary Table S1). Tissue samples were collected from the infected larvae of *S. frugiperda* at 12, 24, 36, 48, 60, 72, and 144 h after treatment with three biological replicates. A total of 10 annotated unigenes were selected randomly and quantified by real-time quantitative PCR (qRT-PCR) with specific primers (Supplementary Table S2) to verify the quality of the mRNA-seq data and expression level. The total RNA of *S. frugiperda* was extracted from samples from LH36, CK36, LH144, and CK144 using the TRIzol method (TaKaRA, Japan). To obtain the first-strand cDNAs, 1 μg of total RNA from the transcriptome samples was reverse-transcribed in a 20-μL reaction system, according to the manufacturer’s instruction (PrimeScript™ RT Reagent Kit, TaKaRa, Japan). qRT-PCR was performed using LightCycler^®^480 SYBR Green I Master (Roche Diagnostics, Basel, Switzerland) and run on the LightCycler^®^480 Real-time PCR system (Roche Diagnostics Ltd.) according to the manufacturer’s instructions. Each reaction was conducted in a 10-μL reaction system with 1 μL cDNA (2 ng/μL), 5 μL SYBR Green I Master (LightCycler^®^480 SYBR Green I Master, Roche Diagnostics Ltd., Lewes, United Kingdom), 0.5 μL/primer, and 3 μL ddH_2_O. The amplification conditions for qRT-PCR were as follows: denaturation at 95°C for 5 min, followed by 40 cycles for 5 s at 95°C, 20 s at 60°C, and 20 s at 72°C. *gapdh* was used as the internal reference gene, and each gene was tested in triplicate. The relative expression levels of the candidate chemosensory genes normalized to the internal control gene were calculated using the 2^−ΔΔCT^ method (Livak and Schmittgen, 2001). Analysis of the relative gene expression data was carried out using real-time quantitative PCR and the 2^−ΔΔCT^ method.

### The effect of signaling pathway inhibitors on antifungal activity in *S. frugiperda*

The third-instar larvae were injected with the Toll inhibitor (BAY 11-7082), Imd inhibitor (parthenolide), JAK/STAT inhibitor mix (20 μM tyrphostin AG 490, Selleck Chemicals, S1143; 5 μM nifuroxazide, Selleck Chemicals, S4182), heat-inactivated *B. bassiana* conidial suspension, and 0.15 mol/L NaCl solution (as the control), with a volume of 1 μL. A total of 60 larvae were injected in each treatment and performed in triplicate. After 24 h, the injected *S. frugiperda* larvae were submerged in *B. bassiana* conidial suspension (10^8^ spores/mL, with 1% penicillin–streptomycin) for 5 s and then reared with fresh corn at 70% humidity and 26°C ± 2°C. The number of dead larvae was recorded every 12 h. A lethal time of 50% (LT_50_) was used to show the resistance of *S. frugiperda* larvae against *B. bassiana*. LT_50_ and confidence intervals of 95% of each treatment were analyzed and calculated using SPSS21.0.

### Data accessibility

The Illumina reads of *S. frugiperda* were submitted to the NCBI Short Archive (SRA) with BioProject PRJNA987447. Their accession numbers are SAMN35982286, SAMN35982287, SAMN35982288, and SAMN35982289.

## Results

### Overview of the transcriptome in *S. frugiperda*

After removing the low-quality reads, trimming off the adapter sequences, sequencing, and a subsequent quality control process, a total of 1,155,833,496 raw reads and 1,111,415,334 clean reads were obtained from those 12 libraries. Furthermore, more than 160 Gb of clean data were obtained. The Q20, Q30, and GC content of each library were over 97.30%, 92.52%, and 43.95%, respectively (Table 1). Unigenes obtained from these transcriptomes were annotated in public databases, including the National Center for Biotechnology Information non-redundant (NCBI-NR) protein, Gene Ontology (GO), and Kyoto Encyclopedia of Genes and Genomes (KEGG) databases. Based on the annotated results of those transcriptomes, the unigene annotation information from LH36vsCK36 and LH144vsCK144 was selected and analyzed.

**TABLE 1:** Summary of the transcriptome of LH36vsCK36 and LH144vsCK144.

Sample	Library	Raw reads	Clean reads	Clean bases (G)	Q30 (%)	GC content (%)
LH36_1	FRAS210259905-1r	97,307,128	91,547,490	13.73	93.33	48.15
LH36_2	FRAS210259906-1r	94,644,560	90,998,532	13.65	93.2	46.61
LH36_3	FRAS210259907-1r	97,734,360	93,820,384	14.07	93.43	45.84
LH144_1	FRAS210259908-1r	93,791,202	91,461,146	13.72	93.01	45.82
LH144_2	FRAS210259909-1r	103,049,876	10,0721,280	15.11	93.63	44.24
LH144_3	FRAS210259910-1r	95,564,128	93,807,964	14.07	93.12	43.95
CK36_1	FRAS210259911-1r	93,592,914	91,906,106	13.79	92.52	44.32
CK36_2	FRAS210259912-1r	89,752,454	86,375,972	12.96	92.71	48.01
CK36_3	FRAS210259913-1r	101,765,194	97,638,290	14.65	93.37	47.7
CK144_1	FRAS210259914-1r	97,647,622	93,568,604	14.04	93.03	47.41
CK144_2	FRAS210259915-1r	87,894,578	82,166,954	12.33	93.16	47.88
CK144_3	FRAS210259916-1r	103,089,480	97,402,612	14.61	93.38	46.85

### Differential gene expression analysis and functional annotation

**FIGURE 1:**
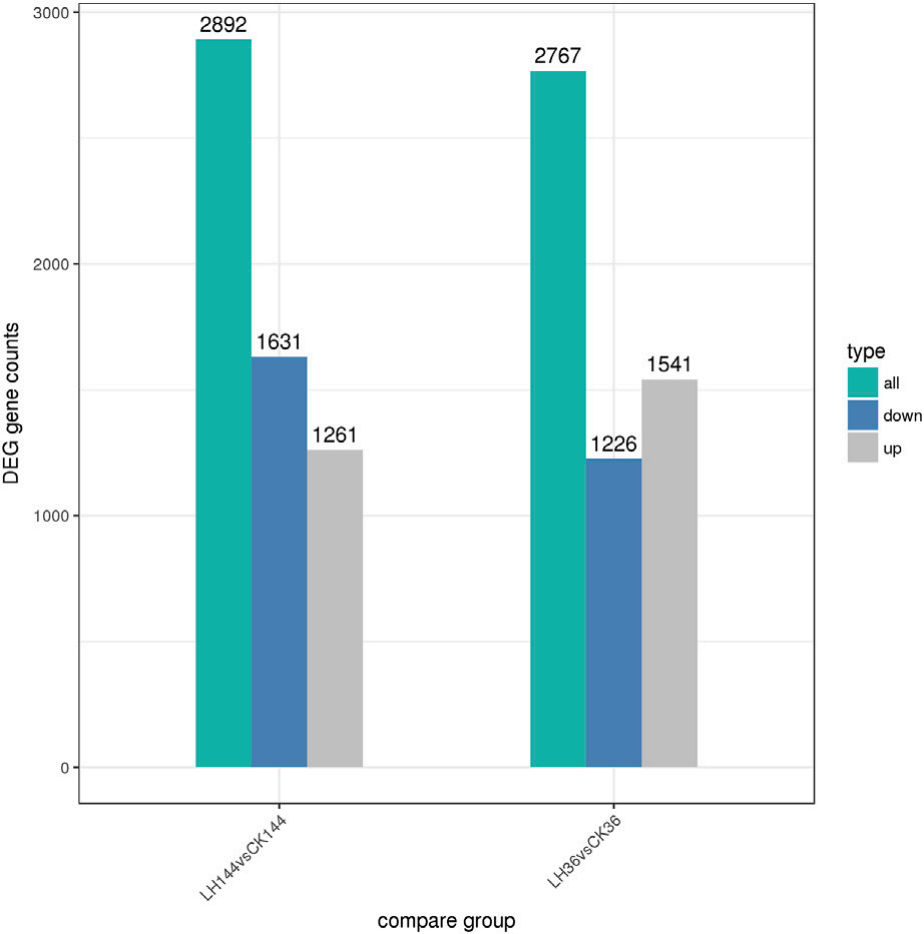
DEGs in LH36vsCK36 and LH144vsCK144.

According to the statistical analysis, 2,767 and 2,892 DEGs were identified in LH36vsCK36 and LH144vsCK144, respectively. In LH36vsCK36, 1,541 DEGs were upregulated and 1,226 DEGs were downregulated, while in LH144vsCK144, 1,261 DEGs were upregulated and 1,631 DEGs were downregulated (Figure 1).

**FIGURE 2:**
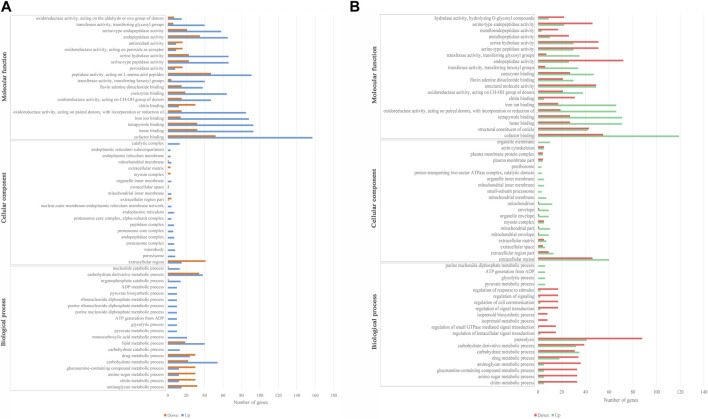
Secondary GO annotation of DEGs. **(A)** Top 20 functional enrichment DEGs in biological process, cellular component, and molecular function in LH36vsCK36; **(B)** top 20 functional enrichment DEGs in biological process, cellular component, and molecular function in LH144vsCK144.

GO enrichment analyses for the DEGs were processed in LH36vsCK36 and LH144vsCK144. A total of 623 and 670 DEGs of LH36vsCK36 and LH144vsCK144 were enriched in GO annotation under three main terms, respectively. In LH36vsCK36, 338, 63, and 222 DEGs were enriched in “biological process,” “cellular component,” and “molecular function,” while in LH144vsCK144, 365, 63, and 241 DEGs were enriched, respectively (Figure 2). A corrected *p*-value <1 was used to screen the significantly enriched GO terms, and the up- and downregulated DEGs in significantly enriched GO terms in LH36vsCK36 and LH144vsCK144 were statistically analyzed.

**FIGURE 3:**
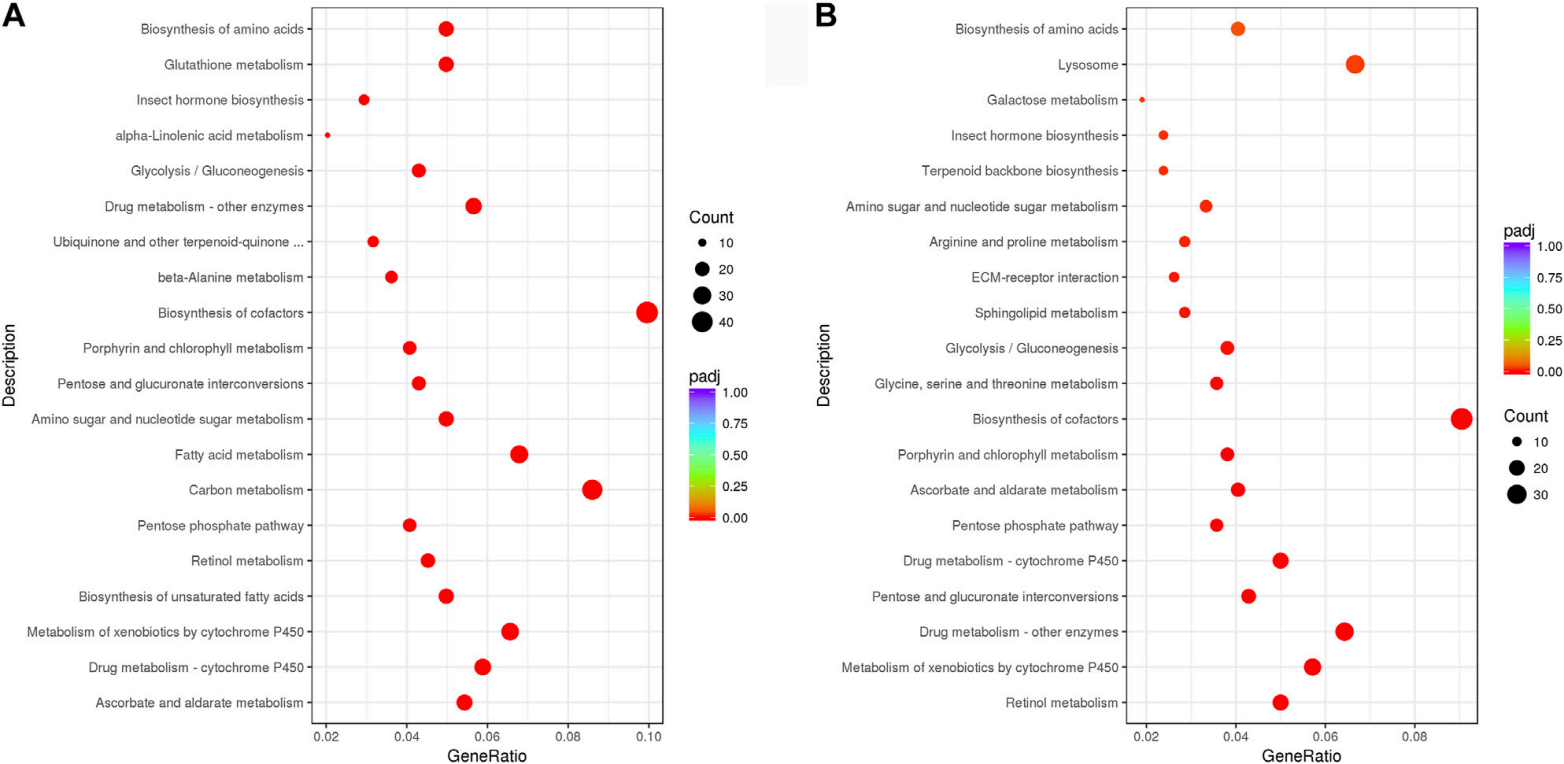
Top 20 pathways of KEGG enrichment of **(A)** LH36vsCK36; **(B)** LH144vsCK144.

From the KEGG enrichment results, the most significant 20 KEGG pathways were selected to draw a scatter plot. In LH36vsCK36, the DEGs were mainly enriched in the “biosynthesis of cofactors” and “carbon metabolism,” followed by “fatty acid metabolism,” “metabolism of xenobiotics by cytochrome P450,” and “drug metabolism—cytochrome P450,” while in LH144vsCK144, the DEGs were mainly enriched in the “biosynthesis of cofactors,” “lysosome,” and “drug metabolism—other enzymes,” followed by “drug metabolism—cytochrome P450,” “metabolism of xenobiotics by cytochrome P450” and “retinol metabolism” (Figure 3).

### Validation of DEG data by qRT-PCR

**FIGURE 4:**
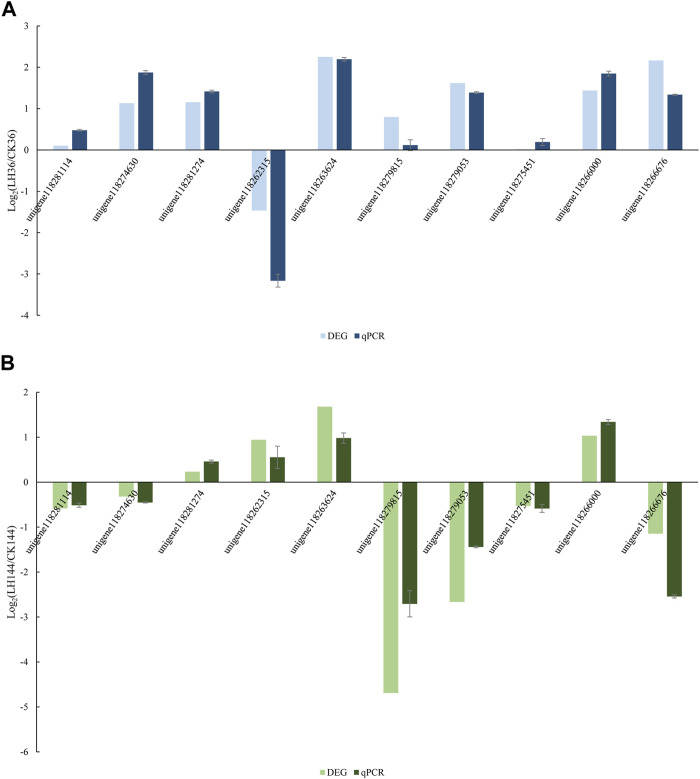
Expression ratios of 10 genes in **(A)** LH36vsCK36; **(B)** LH144vsCK144. Note: The fold changes in the genes were calculated as the log_2_ value of each comparison and are shown on the *x*-axis. Each error bar indicates the standard error with SEMs from the analysis of three replicates (*p* < 0.05).

A total of 10 unigenes were randomly selected for qRT-PCR to confirm the result of the DEG expression using Illumina sequencing in LH36vsCK36 and LH144vsCK144, respectively. Data were presented as log_2_ values of fold changes in gene expression, normalized to *gapdh* relative to each sample. In LH36vsCK36, the qRT-PCR results supported the data obtained by DEG analysis (Figure 4). In LH144vsCK144, the results of the changing trend of qRT-PCR underpinned the reliability of the DEG results.

### Expression patterns of factors related to the Toll signaling pathway in *S. frugiperda* infected with *B*. *bassiana*

**FIGURE 5:**
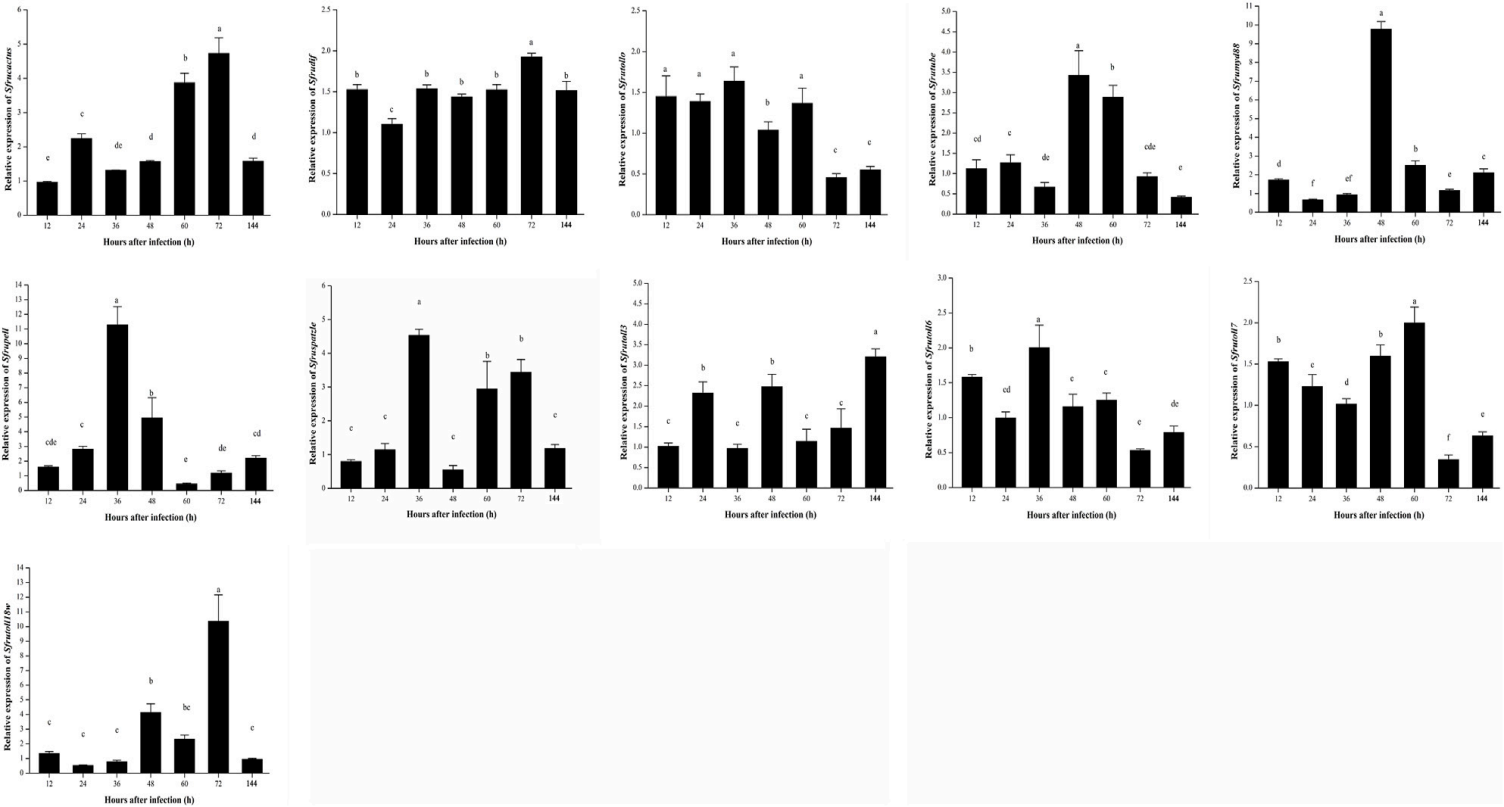
Expression patterns of Toll signaling pathway-related genes in *S. frugiperda* infected by *B. bassiana*.

Sfruspatzle is an extracellular ligand protein of Toll-like receptors (TLRs). The qPCR result revealed that after being infected with *B. bassiana*, the relative expression of Sfruspatzle was slightly upregulated in the early stage of infection, while it was sharply upregulated 48 h after infection (Figure 5). The expression patterns of different Toll receptor genes showed significant differences. After being infected with *B. bassiana*, in *S. frugiperda*, the peaks of relative expression of *Sfrutoll3* occurred at 24, 48, and 144 h; those of *Sfrutoll6* occurred at 12 and 36 h; those of *Sfrutoll7* appeared at 12 and 60 h; those of *Sfrutoll18w* occurred at 48 and 72 h; and *Sfrutollo* showed a high relative expression from 12 to 60 h (Figure 5).

The cytoplasmic signal transduction factors, including *Sfrutube*, *Sfrupell*, *Sfrumyd88*, *Sfrucactus,* and *Sfrudif*, have become the core signal transfer factors in the Toll signaling pathway. The relative expression of *Sfrutube* was significantly upregulated within 48–60 h after infection; the relative expression of *Sfrucactus* was upregulated and peaked within 60–72 h after infection; the relative expression of *Sfrupell* and *Sfrumyd88* peaked at 36 and 48 h, respectively (Figure 5); and there was no significant change in the relative expression of *Sfrudif* throughout the infection stage. These results suggest that there may be different regulatory modes of intracellular signal transduction factors in the Toll signaling pathway of *S. frugiperda* to conduct an immune response against *B. bassiana* infection.

### Expression patterns of factors related to the Imd signaling pathway in *S. frugiperda* infected with *B. bassiana*

**FIGURE 6:**
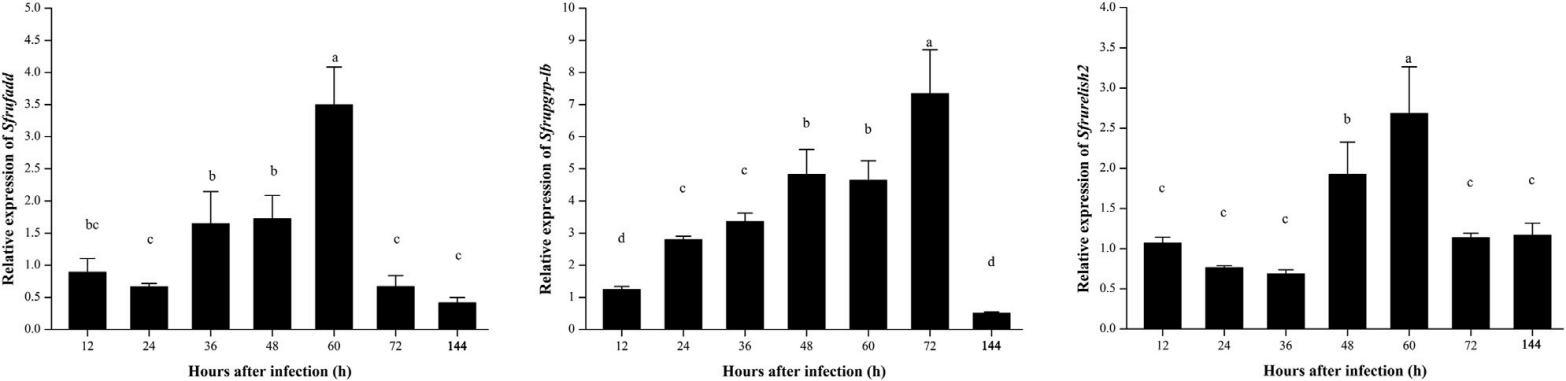
Expression patterns of Imd signaling pathway-related genes in *S. frugiperda* infected by *B. bassiana*.

In this study, the expression patterns of three signal factors associated with Imd signaling pathways were detected after infection by *B. bassiana*. After infection with *B. bassiana*, the expression of related factors of the Imd signaling pathway was changed, and there were significant differences in expression patterns. The relative expression of *Sfrufadd* and *Sfrupgrp-lb* all showed a trend of upregulating and then downregulating, and they reached the peak at 60 and 72 h after infection with *B. bassiana*, respectively, and then downregulated. On the other hand, the relative expression of *Sfrurelish2* was significantly upregulated from 48 to 60 h after being infected with *B. bassiana*, and in other infection stages, it showed no significant differences (Figure 6).

### Expression patterns of factors related to the JAK/STAT signaling pathway in *S. frugiperda* infected with *B. bassiana*

**FIGURE 7:**
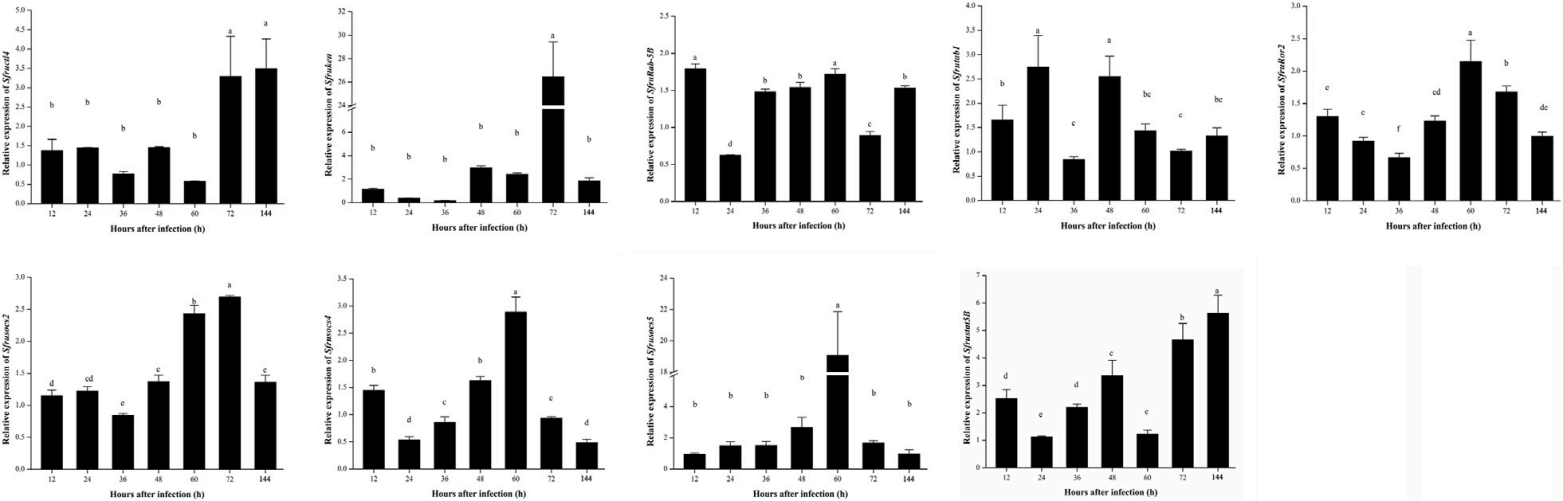
Expression patterns of JAK/STAT signaling pathway-related genes in *S. frugiperda* infected by *B. bassiana*.

In the JAK/STAT signaling pathway, STAT is the core signal transduction factor that is the pattern recognition receptor which can recognize the β-l,3-glucan of fungi (the main component of the fungal cell wall) and lead to the subsequent immune response. When *S. frugiperda* was infected with *B. bassiana*, both *Sfructl4* and *Sfrustat5B* showed a significantly upregulated pattern at 72 and 144 h (Figure 7); *Sfrusocs2*, *Sfrusocs4*, *Sfrusocs5*, *Sfrutab1*, and *Sfruken* are negative regulatory factors of the JAK/STAT signaling pathway, but the mode of regulation remains unclear. After infection with *B. bassiana*, the expression patterns of these five regulatory factors were significantly different. The relative expression levels of *Sfrusocs2* and *Sfrusocs4* presented a similar trend during the whole infection stages; *Sfrusocs5* was significantly upregulated 48 h after infection with *B. bassiana* and peaked at 60 h; and the relative expression levels of *Sfrutab1* were significantly upregulated at 24 and 48 h (Figure 7).

### The effect of signaling pathway inhibitors on the survival rate of *S. frugiperda* infected with *B. bassiana*

**FIGURE 8:**
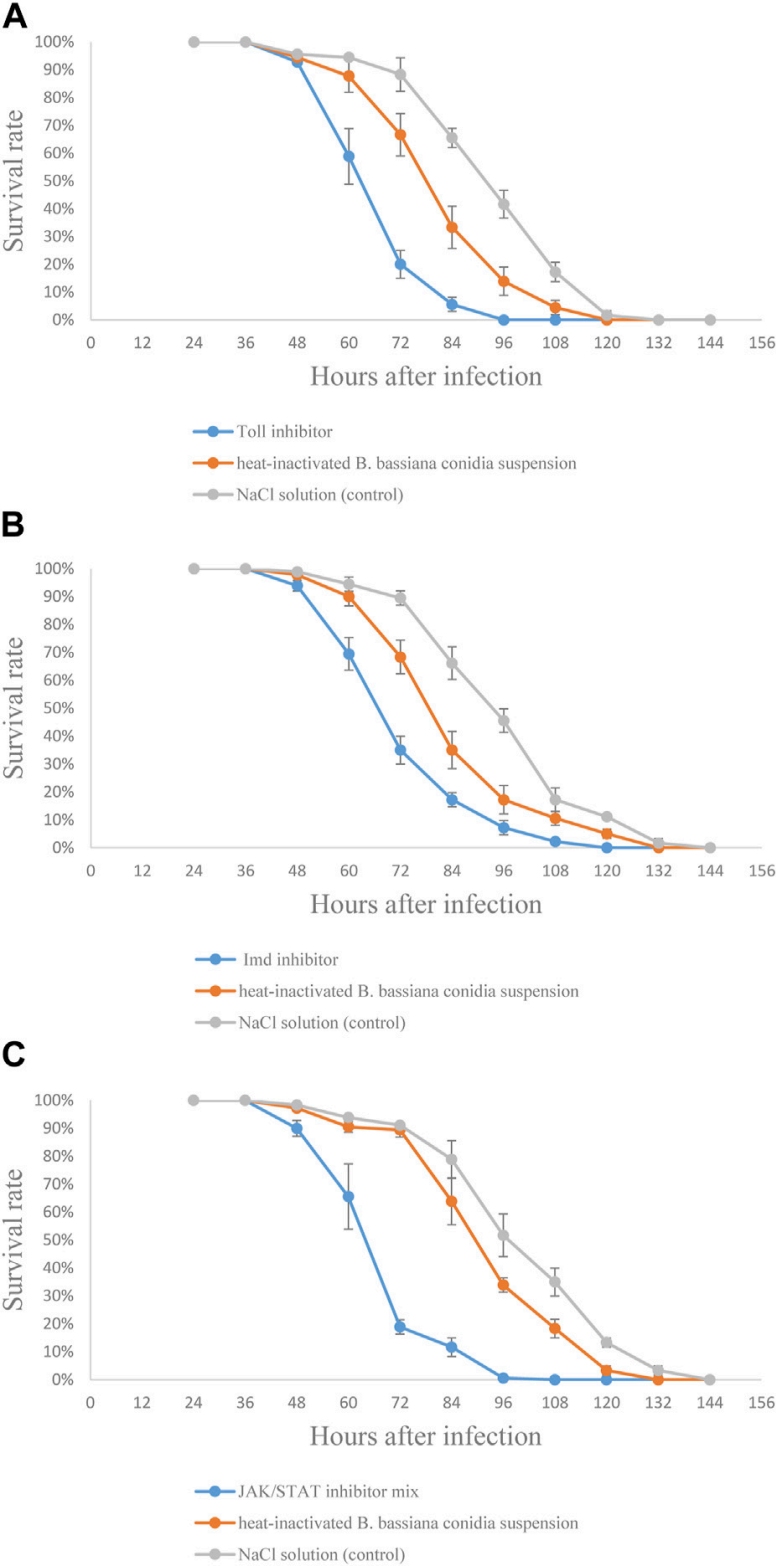
Effects of signaling pathway inhibitors on resistance of *S. frugiperda* larvae against *B. bassiana* infection. **(A)** Test with the Toll signaling pathway inhibitor; **(B)** test with the Imd signaling pathway inhibitor; and **(C)** test with JAK/STAT signaling pathway inhibitor mix.

In order to confirm the functions of Toll, Imd, and JAK/STAT signaling pathways in *S. frugiperda* against *B. bassiana* infection, the effect of signaling pathway inhibitors on the survival rate of *S. frugiperda* infected by *B. bassiana* was tested. The results showed that dead larvae could be found earliest at 48 h and overgrown by the fungus approximately 144 h after injection. In Figure 8, compared to the control group, the survival rate curve was significantly shifted leftward in the group injected with inhibitors (Figure 8). Moreover, the LT_50_ analysis confirmed this observation. As shown in the tables, LT_50_ of the group injected with the Toll inhibitor was 20 h less than that of the control group (Supplementary Table S3); LT_50_ of the group injected with the Imd inhibitor was 21 h less than that of the control group (Supplementary Table S4); and LT_50_ of the group injected with the JAK/STAT inhibitor mix was 24 h less than that of the control group (Supplementary Table S5), indicating that the inhibition of Toll, Imd, and JAK/STAT signaling pathways could accelerate the pathogenicity of *B. bassiana* against *S. frugiperda*.

### Expression patterns of some genes of antibacterial peptides in *S. frugiperda* infected with *B. bassiana*

**FIGURE 9:**
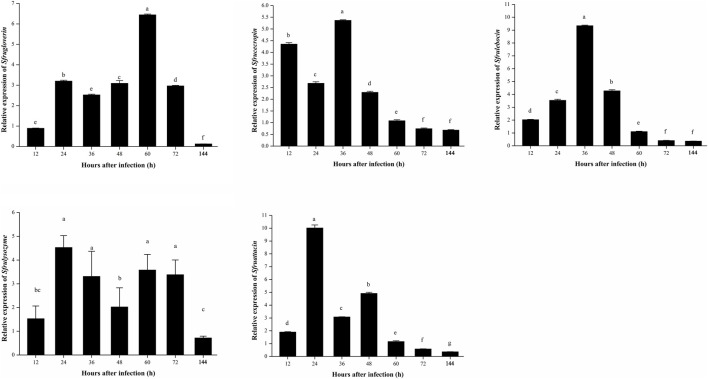
Temporal expression patterns of AMPs in the *S. frugiperda* larvae induced by *B. bassiana*.

The relative expression levels of five genes of antibacterial peptides, *Sfrugloverin*, *Sfrucecropin*, *Sfrulebocin*, *Sfrulysozyme*, and *Sfruattacin*, were analyzed. *Sfrugloverin* and *Sfrulysozyme* showed a similar relative expression trend; they all showed relative high expression from 24 to 72 h after being infected with *B. bassiana*; the relative expression of *Sfrucecropin* and *Sfruattacin* was first upregulated, then downregulated and again upregulated, and again downregulated; and the relative expression of *Sfrulebocin* showed the highest relative expression at 36 h after being infected with *B. bassiana* (Figure 9).

## Discussion

In this study, the transcriptome of *S. frugiperda* infected with *B. bassiana* was analyzed using Illumina HiSeq™ 4000 technology. More than 160 Gb of clean data were obtained. Different DEGs were identified in LH36vsCK36 and LH144vsCK144 libraries, and the upregulated and downregulated genes also showed differences, which might be attributed to the length of time that *S. frugiperda* was infected with *B. bassiana*. This transcriptome sequencing dataset provides a repository for future studies on the interaction mechanism between *S. frugiperda* and *B. bassiana*.

Insects defend against pathogens, such as bacteria, fungi, and viruses, via some evolutionarily conserved signaling pathways, including Toll, Imd, and JAK/STAT (Janeway and Medzhitov, 2002; Nakamoto et al., 2012). These pathways are based on different pattern recognition receptors (PRRs) which recognize characteristic molecules of pathogens and then induce downstream effectors against viral infection (Kumar et al., 2009; Palmer et al., 2018). The Toll signaling pathway is a classical signaling pathway of insect innate immunity. It has been reported that the Toll signaling pathway is activated by fungi and Lys-type peptidoglycan (PG) of Gram-positive bacteria via peptidoglycan recognition protein (PGRP)-SA, PGRP-SD, and Gram-negative binding protein 1 (GNBP1) (Michel et al., 2001; Gobert et al., 2003; Bischoff et al., 2004; Kiyoshi and Shizuo, 2013). In this study, we investigated the role of five Toll-like receptors (TLRs) (*Sfrutollo*, *Sfrutoll3*, *Sfrutoll6*, *Sfrutoll7*, and *Sfrutoll18w*), five cytoplasmic signal transduction factors (*Sfrumyd88*, *Sfrupell*, *Sfrutube*, *Sfrucactus*, and *Sfrudif*), and *Sfruspatzle* in the Toll signaling pathway in *S. frugiperda* against *B. bassiana* infection. The Toll pathway is activated by the activation of Spätzle (Spz), the Toll receptor ligand (Morisato and Anderson, 1994; Schneider et al., 1994).

The relative expression levels of Toll-like receptors (*Sfrutollo*, *Sfrutoll3*, *Sfrutoll6*, *Sfrutoll7*, and *Sfrutoll18w*) and *Sfruspatzle*, *Sfrumyd88*, *Sfrutube*, and *Sfrupell* all showed a significant upregulation at 36 or 48 h after infection, indicating that the Toll signaling pathway of *S. frugiperda* was activated after *B. bassiana* infection and the relative high expression of their corresponding proteins. We speculated that this might be related to the formation of a receptor-proximal oligomeric complex assembled by proteins Spz, Toll receptors and MyD88, Tube, and Pelle, and this complex will further trigger the phosphorylation and regulation of expression of different antimicrobial peptides (AMPs) (Towb et al., 2001). The relative expression levels of *Sfrutollo*, *Sfrutoll6*, and *Sfrutoll7* presented a similar trend, which could be supported by the previous research on *Bombyx mori* (Imamuraand and Yamakawa, 2002; Mita et al., 2004), while the relative expression levels of *Sfrutoll3* and *Sfrutoll18w* showed a difference. The variations in the expression levels of *S. frugiperda* TLRs after *B. bassiana* infection suggested that *S. frugiperda* TLRs might play different roles in antifungal immune responses, and the cytoplasmic signal transduction process of the Toll signaling pathway in *S. frugiperda* had a complex regulatory mechanism, which needed to be further studied.

In order to further verify the role of the Toll signaling pathway in defense against *B. bassiana* in *S. frugiperda*, the antifungal activity *in vivo* was conducted by inhibition assay. The result showed that BAY 11-7082 increased the sensitivity of *S. frugiperda* to *B. bassiana* infection and decreased the antifungal activity of the hemolymph of *S. frugiperda*, suggesting that the Toll signaling pathway plays a crucial role in the synthesis of antifungal substances against *B. bassiana* infection.

The Imd signaling pathway is one of the important signaling pathways in the insect innate immune system. The activated Imd protein acts on TAK 1, which then sends immune signals to IKKs that activate Relish. The activated Relish proteins enter the nucleus to regulate the transcription of target genes such as antimicrobial peptides. It is generally believed that in *Drosophila*, the Imd pathway is activated by meso-diaminopimelic acid (DAP)-type PG of Gram-negative bacteria and some bacilli species (Choe et al., 2002; Gottar et al., 2002; Gobert et al., 2003; Kaneko et al., 2004b), while the results of this experiment show that the Imd signaling pathway of *S. frugiperda* is different from that of *Drosophila* (Zhong et al., 2012). In our study, the relative expression levels of *Sfrufadd*, *Sfrupgrp-lb*, and *Sfrurelish2* all presented a gradual upregulated trend after infection with *B. bassiana*, suggesting that *B. bassiana* could activate the Imd signaling pathway in *S. frugiperda*. In a previous study on *B. mori*, *Staphylococcus aureus*, *Escherichia coli*, and *B. bassiana* were all able to activate the Imd signaling pathway (Cheng et al., 2006). A relatively high expression level of *Sfrulysozyme* was observed at 24 h after infection by *B. bassiana*, which was also found in the previous research on *Galleria mellonella* larvae (Wojda and Jakubowicz, 2007). The variation in the trend of the relative expression level of the rest of the AMP genes in *S. frugiperda* was similar to that in *B. mori* in a previous study (Geng et al., 2016), indicating that they might act as antifungal effectors in *S. frugiperda*. The upregulated expression of antimicrobial peptide genes may be due to the active regulation of *S. frugiperda* antimicrobial peptide expression by *B. bassiana* to inhibit the growth of other bacteria to protect its own nutritional and parasitic requirements (Lee et al., 2005).

The JAK/STAT signaling pathway, which is involved in the regulation of a variety of important physiological processes, is the key signal pathway of immune regulation (Brivanlou et al., 2002b). STAT is the core signal transduction factor of the JAK/STAT signaling pathway, and CTL is a pattern recognition receptor that recognizes fungal β-l,3-glucan (a major component of the fungal cell wall) and guides subsequent immune responses. After infection with *B. bassiana*, the relative expression pattern of *Sfructl4* was synchronized with that of *Sfrustat5B*, indicating that in *S. frugiperda*, the JAK/STAT signaling pathway was activated after infection with *B. bassiana*, and *Sfructl4* and *Sfrustat5B* might play an important role in antifungal immune response (Liu et al., 2015). At the later stage of infection progress, *Sfrustat5B* was upregulated, which might be due to the production of a large number of spores and vegetative hyphae in the late stage of infection, and β-l,3-glucan, the main component of the fungal cell wall, may induce stronger immune responses in *S. frugiperda*. SOCS and Ken are negative regulators of the JAK/STAT signaling pathway. SOCS inhibits the activity of JaK kinase or cytokine receptor (such as STAT) (Cooney, 2002), and Ken selectively regulates the expression of some target genes (Arbouzova et al., 2006). In this study, the relative expression of *Sfrusocs2*, *Sfrusocs4*, *Sfrusocs5*, and *Sfruken* was maintained at a low level in the early stages of infection progress, suggesting that these genes might be involved in the immune response in *S. frugiperda* infected by *B. bassiana*, which indicates that *S. frugiperda* could activate JAK/STAT-mediated immune responses by downregulating the repressors *Sfrusocs2*, *Sfrusocs4*, *Sfrusocs5*, and *Sfruken* (Kausar et al., 2022). The result of the antifungal activity *in vivo* experiment showed that, compared with the control group, the test group (injected with inhibitors) had a lower survival rate and a 20 h reduction in LT_50_. So the JAK/STAT signaling pathway might regulate the synthesis of antifungal substances in *S. frugiperda*, which further indicates that the JAK/STAT signaling pathway plays an important role in the resistance of *S. frugiperda* to *B. bassiana* infection. However, there are also complex regulatory mechanisms in the JAK/STAT signaling pathway, which need to be further studied.

AMPs play an irreplaceable role in the process of innate immunity in insects, and a wide range of antimicrobial peptides display both antibacterial and antifungal functions (Goto et al., 2001; Cerenius et al., 2010; Loof et al., 2011; Browne et al., 2013; Ragland and Criss, 2017; Sheehan et al., 2020). In this study, the relative expression of *Sfrulysozyme* showed a high level from 24 to 72 h after infection by *B. bassiana* (Figure 9); a similar finding was also found in a previous study on *G. mellonella* larvae (Wojda and Jakubowicz, 2007), suggesting that it might be an antifungal function in *S. frugiperda*. The relative expression of *Sfrucecropin*, *Sfruattacin*, *Sfrugloverin*, and *Sfrulebocin* all showed upregulated expression patterns to a varying degree after *S. frugiperda* was infected with *B. bassiana*, indicating that these antimicrobial peptides were involved in the immune response of *S. frugiperda* to *B. bassiana* infection.

In summary, the different expression patterns of Toll, Imd, and JAK/STAT signaling pathway-related genes revealed their different and specific functions in immune signal transduction in *S. frugiperda*. Further studies are needed to investigate the resistance mechanism of *S. frugiperda* to pathogenic microorganisms. This study not only lays a solid foundation for the research and development of new fungal agents to control *S. frugiperda* and other pests but also provides an important scientific basis for the development and use of new biocontrol agents.

## Data Availability

The datasets presented in this study can be found in online repositories. The names of the repository/repositories and accession number(s) can be found in the article/Supplementary Material.
