# Supplementary material for: Induced expression modes of genes related to Toll, Imd, and JAK/STAT signaling pathway-mediated immune response in Spodoptera frugiperda infected with Beauveria bassiana
Source: Front Physiol. 2023 Aug 24;14:1249662. doi: 10.3389/fphys.2023.1249662 (PMC10484109; doi:10.3389/fphys.2023.1249662)
Supplement: Supplementary file 1 [file Table1.DOCX]

Supplementary Table 1 Primers of signaling pathway related genes used in qRT-PCR

| Gene | Primer sequences (5’-3’) | S/AS |
| --- | --- | --- |
| *gapdh* | ACCAACTGTCTCGCTCCTCT | S |
|  | GAGGGTCCGTCAACAGTCTT | AS |
| *Sfufadd* | GGTAGTTTCTGGCGTGAT | S |
|  | ATCCTTTCTACGGCTTTT | AS |
| *Sfupgrp lb* | GGACGATAGAGGTTGAAGAA | S |
|  | TGGAAAGTTGTAGGGATGG | AS |
| *Sfurelish2* | GTTGGGCGATTTGTGC | S |
|  | ACGCTTGGTTGGCTCT | AS |
| *Sfuctl4* | GCATTTCGTTGCGACTAC | S |
|  | TGCTTCATCTCAGCCTCT | AS |
| *Sfuken* | GTCACGCCTCACCGAAA | S |
|  | CCGAGCACTGGCAACTG | AS |
| *Sfucecropin* | CGTGTCGTATCACCAGAG | S |
|  | TGAAGAATTTCCACCTTG | AS |
| *SfuRab-5B* | TCGCTCTCTCGCTTCCA | S |
|  | ATACCGCTGTTTTGCAC | AS |
| *SfuRor2* | GATGTGTGGGCTTATGGGG | S |
|  | ACCTGGGTGTGTTGTCTGG | AS |
| *Sfusocs2* | TAATGCCTAATCTTGTCC | S |
|  | TGTATGTGTGAGTGCCGA | AS |
| *Sfusocs4* | TCCTCCGAGACTCTGCC | S |
|  | CGGGGTCCTTGTAATGC | AS |
| *Sfusocs5* | TATTCTCTGTGTGTCCTTCCG | S |
|  | ACCTATTTAGTGGCCTTGCTA | AS |
| *Sfustat5B* | ACTGTCCGTTTACTGGT | S |
|  | TGGGTCTCGCTTTTTAG | AS |
| *Sfutab1* | GAGACACAGGTATCCCAAA | S |
|  | TCAGCACAGCATTATCATC | AS |
| *Sfucactus* | ATTACTTCCTTGCCGACACCAC | S |
|  | TTACCCTTCGCTTCAGTCACAT | AS |
| *Sfudif* | AGGCGGGACCCAAGTTATCT | S |
|  | GTACGCTGGCGTCTCGAAAG | AS |
| *Sfugloverin* | AACGGAGACAGCAGCAACC | S |
|  | CCATCACCATTTAGGGACA | AS |
| *Sfulysozyme* | CAAAGATTGGTGCAAGAAGG | S |
|  | AATAAGAGAAAGAGGTCGGC | AS |
| *Sfumyd88* | TAGAATGAGTGCTGAAGGG | S |
|  | TGAGAAGGAAGTCTGGCGA | AS |
| *Sfupell* | CGCTTGGAGGTCCAGAAGC | S |
|  | GCCGTGAATAAGGGGGGTA | AS |
| *Sfuspatzle* | TAGGACCTGTTGTCACCCACA | S |
|  | TCAGAATACCGAATGCTTTAT | AS |
| *Sfutoll3* | GAGGGACTTTCTTGTGGG | S |
|  | TTAGGTCTTCTCTGCGGG | AS |
| *Sfutoll6* | GTTTACAGATTTACCGTCCCT | S |
|  | TATTCGTTAGTTGGTTAGCAT | AS |
| *Sfutoll7* | TATGGATGCAACCGAGGTA | S |
|  | CATTCAAGCCAGCAAAAGT | AS |
| *Sfutoll18w* | TCTGGTTTGATTACGCTTTT | S |
|  | TGGAGTTCATTTTGGATTTT | AS |
| *Sfutollo* | CAAAGTAGTCTCGCTCCG | S |
|  | TTTCTTAATTCCCGCAGT | AS |
| *Sfutube* | TAAAAGGCACAGCAGAAGG | S |
|  | GCCAAGCCAAAGTCACAAA | AS |
| *Sfuattacin* | TGTTCAGAAACAAAGACA | S |
|  | TAAATCCAGTAGAAGGCT | AS |
| *Sfulebocin* | CCAAACAGGAGAACAGCG | S |
|  | ATGAGGATTGAAAGGCGG | AS |
