# Supplementary material for: Induced expression modes of genes related to Toll, Imd, and JAK/STAT signaling pathway-mediated immune response in Spodoptera frugiperda infected with Beauveria bassiana
Source: Front Physiol. 2023 Aug 24;14:1249662. doi: 10.3389/fphys.2023.1249662 (PMC10484109; doi:10.3389/fphys.2023.1249662)
Supplement: Supplementary file 2 [file Table2.DOCX]

Supplementary Table 2 Primers used in qRT-PCR

| Gene ID | Primer sequences (5’-3’) | S/AS |
| --- | --- | --- |
| 118281114 | CGCCGGTAAGGGAAAGAT | S |
|  | TCCACGCCTGGGATGTTG | AS |
| 118274630 | TGTTCGTGTTGGTCGCTT | S |
|  | GTCCTTGATGGCCTTGTT | AS |
| 118281274 | TACGCTCTGTCTCGCAAG | S |
|  | TCAGGGCCAAACATAATT | AS |
| 118262315 | CGGAGAACTCAAACACGG | S |
|  | CAAATAAGGATTGGACGG | AS |
| 118263624 | AATCTGAAGTGACCGTGG | S |
|  | CCGTAAAATCTCTTGCCC | AS |
| 118279815 | GAGATGCGGTGAAGTTTG | S |
|  | CTTGGTGTCTGCTGCTGG | AS |
| 118279053 | ACAAACCATCAAGCTCAA | S |
|  | CTCACTCGTCACTCGCAC | AS |
| 118275451 | TTGATGGAAGAGGTGTGC | S |
|  | CTTGGAGTGGAAGGAGTG | AS |
| 118266000 | TTGGGCTAAAGAATGAAA | S |
|  | GCAGGAGCAGAGAACGAT | AS |
| 118266676 | AGAAAGGCGTGAAAGTAG | S |
|  | TAGAGCGTGGATAACAAA | AS |
