# Supplementary material for: Induced expression modes of genes related to Toll, Imd, and JAK/STAT signaling pathway-mediated immune response in Spodoptera frugiperda infected with Beauveria bassiana
Source: Front Physiol. 2023 Aug 24;14:1249662. doi: 10.3389/fphys.2023.1249662 (PMC10484109; doi:10.3389/fphys.2023.1249662)
Supplement: Supplementary file 3 [file Table5.DOCX]

Supplementary Table 5 The Probit test of LT_50_ of JaK/STAT signaling pathway inhibitor treatment.

| Treatment | Fitted equation | LT_50_ | 95% confidence intervals | |
| --- | --- | --- | --- | --- |
| JaK/STAT signaling pathway inhibitor | P = −5.104 + 0.079x | 64.626 | 62.262 | 66.97 |
| Heat-inactivated *B. bassiana* suspension | P = −4.872 + 0.055x | 89.325 | 86.511 | 92.158 |
| Control | P = −4.783 + 0.049x | 97.614 | 94.642 | 100.622 |
