# Supplementary material for: Induced expression modes of genes related to Toll, Imd, and JAK/STAT signaling pathway-mediated immune response in Spodoptera frugiperda infected with Beauveria bassiana
Source: Front Physiol. 2023 Aug 24;14:1249662. doi: 10.3389/fphys.2023.1249662 (PMC10484109; doi:10.3389/fphys.2023.1249662)
Supplement: Supplementary file 4 [file Table3.DOCX]

Supplementary Table 3 The Probit test of LT_50_ of Toll signaling pathway inhibitor treatment.

| Treatment | Fitted equation | LT_50_ | 95% confidence intervals | |
| --- | --- | --- | --- | --- |
| Toll signaling pathway inhibitor | P = −5.724 + 0.090x | 63.422 | 61.208 | 65.618 |
| Heat-inactivated *B. bassiana* suspension | P = −3.859 + 0.046x | 83.423 | 78.384 | 88.608 |
| Control | P = −4.999 + 0.055x | 90.09 | 87.302 | 92.908 |
