# Supplementary material for: Induced expression modes of genes related to Toll, Imd, and JAK/STAT signaling pathway-mediated immune response in Spodoptera frugiperda infected with Beauveria bassiana
Source: Front Physiol. 2023 Aug 24;14:1249662. doi: 10.3389/fphys.2023.1249662 (PMC10484109; doi:10.3389/fphys.2023.1249662)
Supplement: Supplementary file 5 [file Table4.DOCX]

Supplementary Table 4 The Probit test of LT_50_ of Imd signaling pathway inhibitor treatment.

| Treatment | Fitted equation | LT_50_ | 95% confidence intervals | |
| --- | --- | --- | --- | --- |
| Imd signaling pathway inhibitor | P = −4.386 + 0.063x | 69.428 | 66.782 | 72.04 |
| Heat-inactivated *B. bassiana* suspension | P = −4.410 + 0.054x | 81.303 | 78.456 | 84.12 |
| Control | P = −4.880 + 0.053x | 92.935 | 90.061 | 95.814 |
